# Supplementary material for: Content-rich biological network constructed by mining PubMed abstracts
Source: BMC Bioinformatics. 2004 Oct 8;5:147. doi: 10.1186/1471-2105-5-147 (PMC528731; doi:10.1186/1471-2105-5-147)
Supplement: Additional File 2 — The original results of the above study (non-essential files are deleted to keep the file size under the limit set by BMC bioinformatics). [file 1471-2105-5-147-S2.bz2 › chilibotAdditionalFile2/dip20/600ID10092459E10979/html/PHES_PHET.html]

 


 **PHES** and **PHET** 
  
Found 54 abstracts in PubMed, retrieved 20.  
 

 What does Google say? 
 PDF only 
| .edu only 

---

**Interactive relationship** (e.g. stimulation, inhibition, etc)

**Neutral relationship**- The conformation of PheOH AMP and the network of interactions of its AMP moiety with PheRS  [ **PHET** ]   [ **PHES** ]  are reminiscent of the other class II synthetases.  Ref: 10092459 J Mol Biol, 1999
- The recognition of Phe by PheRS  [ **PHET** ]   [ **PHES** ]  is achieved through a mixture of multiple van der Waals interactions and hydrogen bonds.  Ref: 10092459 J Mol Biol, 1999
- In prokaryotes, the alpha and beta subunits of pheRS  [ **PHET** ]   [ **PHES** ]  are encoded by the genes  **pheS**  and  **pheT** , respectively.  Ref: 11281723 Protein Expr Purif, 2001
- Tn5luxAB was localized to a fragment of chromosomal DNA encoding the thrS, infC, rpmI, rplT,  **pheS**  and  **pheT**  genes, with Tn5 inserted in the 3 end of  **pheS** .  Ref: 12583892 FEMS Microbiol Lett, 2003
- A sulfate ion, which was identified on the protein surface, may mediate the interactions of PheRS  [ **PHET** ]   [ **PHES** ]  with DNA.  Ref: 11679717 Acta Crystallogr D Biol Crystallogr, 2001
- Our results indicate that while OA may be capable of weakly inhibiting PheRS  [ **PHET** ]   [ **PHES** ] , the OA PheRS  [ **PHET** ]   [ **PHES** ]  complex cannot adopt the same conformation as does FAMP PheRS  [ **PHET** ]   [ **PHES** ] , contrary to previous assumptions.  Ref: 10447951 J Med Chem, 1999

**Non-interactive relationship** (e.g. studied together, co-existance, homology, etc.)

- Unlike the catalytic alpha subunit, the beta subunit of heterodimeric alphabeta 2 phenylalanyl tRNA synthetase PheRS  [ **PHET** ]   [ **PHES** ]  has no invariant functional amino acids directly involved in the aminoacylation process as it is evident from the crystal structure of the T. thermophilus enzyme complexed with tRNAPhe.  Ref: 10049785 Biochem Biophys Res Commun, 1999
- The h mt TrpRS shows kinetic properties similar to human mitochondrial phenylalanyl tRNA synthetase h mt PheRS  [ **PHET** ]   [ **PHES** ] , and h mt TrpRS is not induced by interferon gamma as is hTrpRS.  Ref: 10828066 J Biol Chem, 2000
- Evolutionary origins of this small monomeric human mtPheRS are unknown implications are that this enzyme is a result of the simplification of the more complex alphabeta 2 bacterial PheRS  [ **PHET** ]   [ **PHES** ]  in which specific functional regions were retained.  Ref: 10329163 J Mol Biol, 1999
- In this study, we incorporate para bromophenylalanine p Br phe into a model target protein, mouse dihydrofolate reductase DHFR, by using a bacterial phenylalanyl tRNA synthetase PheRS  [ **PHET** ]   [ **PHES** ]  variant with relaxed substrate specificity.  Ref: 10664452 FEBS Lett, 2000
- We describe the high level overexpression and purification of recombinant S. aureus pheRS  [ **PHET** ]   [ **PHES** ]  using  **pheS**  and  **pheT**  genes as part of an artificial operon in Escherichia coli.  Ref: 11281723 Protein Expr Purif, 2001
- Two ORFs with similarity to non archaeal PheRSs alpha subunits had previously been found in the genome sequence, but these results show that only one of them, MT742, is part of the active PheRS  [ **PHET** ]   [ **PHES** ] .  Ref: 10575359 Biochimie, 1999
- In this paper, we describe a computational protocol HierDock for predicting the relative energies of binding of phenylalanine analogues to phenylalanyl tRNA synthetase PheRS  [ **PHET** ]   [ **PHES** ] .  Ref: 12452720 J Am Chem Soc, 2002
- The structural similarity between PheRS  [ **PHET** ]   [ **PHES** ]  and histidyl tRNA synthetase extends to the amino acid binding site, which is normally unique for each enzyme.  Ref: 10092459 J Mol Biol, 1999
- The interaction of Thermus thermophilus phenylalanyl tRNA synthetase PheRS  [ **PHET** ]   [ **PHES** ]  with the 3 ; terminal nucleotide of tRNAPhe has been studied by affinity labeling to solve the problem arising from X ray crystallographic study the binding sites of phenylalanine and the 3 ; terminal nucleotide base were revealed to be identical in the crystal structures of PheRS  [ **PHET** ]   [ **PHES** ]  complexed with the substrates.  Ref: 11092959 Biochemistry Mosc , 2000
- The level of PheRS  [ **PHET** ]   [ **PHES** ]  alpha subunit like mRNA is regulated during differentiation but not during cell cycle progression.  Ref: 10375616 Gene, 1999
- The complex structures suggest that the PheRS  [ **PHET** ]   [ **PHES** ]  beta subunit may affect the first step of the reaction formation of phenylalanyl adenylate through the metal mediated conserved alpha beta subunit interface.  Ref: 10092459 J Mol Biol, 1999
- For comparative analysis we also report a procedure for the purification of native pheRS  [ **PHET** ]   [ **PHES** ]  from S. aureus Oxford Strain and demonstrate that Michaelis Menten parameters for both recombinant and native enzyme, at least for phenylalanine tRNA aminoacylation are comparable.  Ref: 11281723 Protein Expr Purif, 2001
- Coexpression of the mutant PheRS  [ **PHET** ]   [ **PHES** ]  and DHFR in a phenylalanine auxotrophic Escherichia coli host strain grown in p Br phe supplemented minimal medium resulted in 88% replacement of phenylalanine residues by p Br phe.  Ref: 10664452 FEBS Lett, 2000
- The modeling of tyrosine in the active site of PheRS  [ **PHET** ]   [ **PHES** ]  revealed no apparent close contacts between tyrosine and the PheRS  [ **PHET** ]   [ **PHES** ]  residues.  Ref: 10092459 J Mol Biol, 1999
- Crystals of native PheRS  [ **PHET** ]   [ **PHES** ]  were soaked in a solution containing phenylalanine and ATP in the presence of Mn 2 ions.  Ref: 11679717 Acta Crystallogr D Biol Crystallogr, 2001
- The dissociation constants for the eEF1A.GDP.tRNA and eEF1A.GDP.tRNAPhe.PheRS  [ **PHET** ]   [ **PHES** ]  complexes were found to be 20 nm and 9 nm, respectively.  Ref: 12354112 Eur J Biochem, 2002
- Functional activity of the covalent complex of PheRS  [ **PHET** ]   [ **PHES** ]  with the s4U monosubstituted transcript has been shown by aminoacylation of 60% of the enzyme cross linked tRNA.  Ref: 9613588 FEBS Lett, 1998
- Phenylalanyl tRNA synthetase pheRS  [ **PHET** ]   [ **PHES** ]  is unique among aminoacyl tRNA synthetases in that it is a heterotetrameric enzyme composed of two alpha subunits and two larger beta subunits.  Ref: 11281723 Protein Expr Purif, 2001
- This, in turn, suggests that the previously observed antagonistic effects of aspartame and piroxicam are more likely due to their prevention of OA binding to human serum albumin than to PheRS  [ **PHET** ]   [ **PHES** ] , which is in agreement with binding studies as well as with preliminary simulations performed in our laboratory.  Ref: 10447951 J Med Chem, 1999
- Comparative analysis of the cross linked products in the absence and in the presence of small substrates revealed ATP and aminoacyl adenylate to effect the interaction of the tRNAPhe acceptor end with PheRS  [ **PHET** ]   [ **PHES** ] .  Ref: 11092959 Biochemistry Mosc , 2000
- Human phenylalanyl tRNA synthetase PheRS  [ **PHET** ]   [ **PHES** ]  was cloned and expressed in Escherichia coli.  Ref: 11858721 Protein Expr Purif, 2002
- In this report we describe the isolation of a DNA fragment 3.52 kb containing the  **pheS**  and  **pheT**  genes from a Staphylococcus aureus WCUH29 genomic DNA library.  Ref: 11281723 Protein Expr Purif, 2001
- The data obtained, combined with kinetic data on aminoacylation, clearly show that PheRS  [ **PHET** ]   [ **PHES** ]  exhibits more tRNA selectivity at the level of binding than at the level of catalysis.  Ref: 12382236 J Mol Recognit< MedlineTA>J Mol Recognit, 2001
- Here we present the data on association of the eEF1A.GDP.tRNA complex with phenylalanyl tRNA synthetase PheRS  [ **PHET** ]   [ **PHES** ] , e.g. the formation of the above quaternary complex detected by the gel retardation and surface plasmon resonance techniques.  Ref: 12354112 Eur J Biochem, 2002
- The extent of tRNA recognition at the level of binding by Thermus thermophilus phenylalanyl tRNA synthetase PheRS  [ **PHET** ]   [ **PHES** ] , one of the most complex class II synthetases, has been studied by independent measurements of the enzyme association with wild type and mutant tRNA Phe s as well as with non cognate tRNAs.  Ref: 12382236 J Mol Recognit< MedlineTA>J Mol Recognit, 2002
- Both analogs have been cross linked to Thermus thermophilus phenylalanyl tRNA synthetase PheRS  [ **PHET** ]   [ **PHES** ]  and the specificity of the cross linking has been demonstrated.  Ref: 9613588 FEBS Lett, 1998
- Detailed analysis indicates that unlike the alphabeta 2 structure of the prokaryotic and eukaryotic cytoplasmic forms of PheRS  [ **PHET** ]   [ **PHES** ] , the human mtPheRS consists of a single polypeptide chain.  Ref: 10329163 J Mol Biol, 1999
- Starting with the crystal structure of Thermus thermophilus PheRS  [ **PHET** ]   [ **PHES** ]  without bound ligand, HierDock predicts the binding site of phenylalanine Phe within 1.1 A of that revealed by the crystal structure of PheRS  [ **PHET** ]   [ **PHES** ]  cocrystallized with Phe.  Ref: 12452720 J Am Chem Soc, 2002
- The results suggest specific binding of the 3 ; terminal nucleotide of tRNAPhe by the catalytic subunit of PheRS  [ **PHET** ]   [ **PHES** ]  in the absence of other substrates.  Ref: 11092959 Biochemistry Mosc , 2000
- Among other mechanisms of toxicity, it has been suggested that OA inhibits phenylalanyl tRNA synthetase PheRS  [ **PHET** ]   [ **PHES** ] , thereby reducing protein synthesis.  Ref: 10447951 J Med Chem, 1999
- This is the first report of the cloning of the full length cDNA for the murine PheRS  [ **PHET** ]   [ **PHES** ]  regulatory alpha subunit like protein.  Ref: 10375616 Gene, 1999
- In particular, it is suggested that the monomeric PheRS  [ **PHET** ]   [ **PHES** ]  from the yeast mitochondrion is a chimera of the alpha and beta chains of the standard tetrameric protein.  Ref: 12572618 Mol Biol Evol, 1998
- The predicted amino acid sequence is 589 amino acids and shares 45% identity with the yeast cytoplasmic phenylalanyl tRNA synthetase PheRS  [ **PHET** ]   [ **PHES** ]  regulatory alpha subunit.  Ref: 10375616 Gene, 1999
- In addition, PheRS  [ **PHET** ]   [ **PHES** ]  residues Trpalpha149, Seralpha180, Hisalpha178, Argalpha204, Glnalpha218, and Glualpha220 form a total of seven hydrogen bonds with the main chain atoms of Phe.  Ref: 10092459 J Mol Biol, 1999
- We also revealed a direct interaction of PheRS  [ **PHET** ]   [ **PHES** ]  with eEF1A in the absence of tRNAPhe Kd = 21 nm.  Ref: 12354112 Eur J Biochem, 2002
- This result implies that the proofreading mechanism against activated tyrosine, rather than direct recognition, may play the major role in the PheRS  [ **PHET** ]   [ **PHES** ]  specificity.  Ref: 10092459 J Mol Biol, 1999
- A few backbone mediated contacts of PheRS  [ **PHET** ]   [ **PHES** ]  with the acceptor and anticodon stems revealed in the crystal structure do not contribute to tRNA Phe discrimination, their role being limited to stabilization of the complex.  Ref: 12382236 J Mol Recognit< MedlineTA>J Mol Recognit, 1999
- Identification, cloning, and expression of a functional phenylalanyl tRNA synthetase pheRS  [ **PHET** ]   [ **PHES** ]  from Staphylococcus aureus.  Ref: 11281723 Protein Expr Purif, 2001
- Using consensus sequences derived from conserved regions of the alpha and beta subunits from bacterial PheRS  [ **PHET** ]   [ **PHES** ] , two partially sequenced cDNA clones were identified.  Ref: 10329163 J Mol Biol, 1999
- Aminoacylation activity of the overproduced human PheRS  [ **PHET** ]   [ **PHES** ]  in the crude bacterial extract is two orders of magnitude higher than the corresponding activity in human placenta and the yield of the recombinant enzyme overproduced in E. coli is five times higher.  Ref: 11858721 Protein Expr Purif, 2002
- The calculated binding energies of Phe analogues in PheRS  [ **PHET** ]   [ **PHES** ] , using HierDock, correlate well with the translational activities of the same analogues in Escherichia coli.  Ref: 12452720 J Am Chem Soc, 2002
- Remarkably, both rates of aminoacyl transfer and amino acid specificities are greater for RNA 77 than measured for protein PheRS  [ **PHET** ]   [ **PHES** ] .  Ref: 10318907 Proc Natl Acad Sci U S A, 1999
- Visible conformational changes were detected in the active site area adjacent to the position of the Phe AMP, compared with the structure of PheRS  [ **PHET** ]   [ **PHES** ]  complexed with a synthetic adenylate analogue phenylalaninyl adenylate.  Ref: 11679717 Acta Crystallogr D Biol Crystallogr, 2001
- Both Phe and PheOH AMP are engulfed in the active site cleft of the catalytic alpha subunit of PheRS  [ **PHET** ]   [ **PHES** ] , and neither makes contact with the PheRS  [ **PHET** ]   [ **PHES** ]  beta subunit.  Ref: 10092459 J Mol Biol, 1999
- We cloned, sequenced, and expressed human PheRS  [ **PHET** ]   [ **PHES** ] .  Ref: 10049785 Biochem Biophys Res Commun, 1999
- HierDock identifies p fluorophenylalanine and 3 thienylalanine as especially good substrates for PheRS  [ **PHET** ]   [ **PHES** ] , in agreement with experiment.  Ref: 12452720 J Am Chem Soc, 2002
- The crystal structure of phenylalanyl tRNA synthetase PheRS  [ **PHET** ]   [ **PHES** ]  from Thermus thermophilus, a class II aminoacyl tRNA synthetase, complexed with phenylalanyl adenylate Phe AMP was determined at 2.6 A resolution.  Ref: 11679717 Acta Crystallogr D Biol Crystallogr, 2001
- The N terminal 314 amino acid residues appear to be analogous to the alpha subunit of the prokaryotic PheRS  [ **PHET** ]   [ **PHES** ] , while the C terminal 100 amino acid residues correspond to a region of the beta subunit known to interact with the anticodon of tRNAPhe.  Ref: 10329163 J Mol Biol, 1999
- Three different types of alphabeta 2 heterodimers of human PheRS  [ **PHET** ]   [ **PHES** ]  carrying HT at the N terminus of either of two alpha or beta subunits or simultaneously on both of them were overproduced and purified.  Ref: 11858721 Protein Expr Purif, 2002
- The highest affinity of T. thermophilus PheRS  [ **PHET** ]   [ **PHES** ]  for cognate tRNA, observed for synthetase tRNA complexes, results in 100 3000 fold binding discrimination against non cognate tRNAs.  Ref: 12382236 J Mol Recognit< MedlineTA>J Mol Recognit, 2002
- Two of the R M systems, NmeBI and NmeDI, were located at homologous positions between the phenylalanyl tRNA synthetase genes  **pheS**  and  **pheT** , which appeared to be a preferential target for the insertion of foreign DNA in meningococci.  Ref: 10671450 J Bacteriol, 2000
- Using the crystal structure of PheRS  [ **PHET** ]   [ **PHES** ]  from Thermusthermophilus, we have modeled its interactions with OA as well as with phenylalanyl adenylate FAMP, the high affinity intermediate substrate of PheRS  [ **PHET** ]   [ **PHES** ] .  Ref: 10447951 J Med Chem, 1999
- Comparisons with the sequences of PheRS  [ **PHET** ]   [ **PHES** ]  from yeast and Drosophila mitochondria indicate they are 42 % and 51 % identical with the human mtPheRS, respectively.  Ref: 10329163 J Mol Biol, 1999
- The crystal structures of Thermus thermophilus phenylalanyl tRNA synthetase PheRS  [ **PHET** ]   [ **PHES** ]  complexed with phenylalanine and phenylalaninyl adenylate PheOH AMP, the synthetic analogue of phenylalanyl adenylate, have been determined at 2.7A and 2.5A resolution, respectively.  Ref: 10092459 J Mol Biol, 1999
- The correct positioning of the 3 ; terminal nucleotide of tRNAPhe corresponding to the structure of the productive complex with PheRS  [ **PHET** ]   [ **PHES** ]  is therefore promoted only in the presence of all three substrates.  Ref: 11092959 Biochemistry Mosc , 2000
